# Use of D-dimer to Screen for Cerebral Pathology in ED Patients with Non-traumatic Headache and Normal Neurological Exam

**DOI:** 10.5811/westjem.48604

**Published:** 2026-02-22

**Authors:** Cenker Eken, Mustafa Serinken, Faruk Güngör, Ömer Akdağ

**Affiliations:** * Denipollife Hospital, Department of Emergency Medicine, Denizli, Türkiye; † ASV Yaşam Hospital, Department of Emergency Medicine, Antalya, Türkiye; ‡ Isparta State Hospital, Department of Emergency Medicine, Isparta, Türkiye

## Abstract

**Introduction:**

Our goal in this study was to evaluate the diagnostic utility of bedside D-dimer testing for identifying secondary headache due to intracranial pathology among patients presenting to the emergency department (ED) with non-traumatic headache and no neurological deficits.

**Methods:**

We conducted this prospective, multicenter, cross-sectional study across six tertiary care EDs in Türkiye. Adult patients presenting with non-traumatic headache and no neurological deficits who underwent cranial computed tomography (CT) based on clinical suspicion for intracranial pathology were enrolled. Exclusion criteria were recent trauma, pregnancy, fever, hematologic conditions, and known intracranial pathology. We measured bedside D-dimer using a D-dimer assay with a predefined threshold of 500 nanograms per milliliter. The primary outcome was secondary headache related to intracranial pathologies as determined on the index CT and additional tests as needed or during one-month follow-up.

**Results:**

Of the 3,279 patients screened, 1,522 were included in the final analysis. Secondary headache due to intracranial pathology was identified in 57 patients (3.7%). The most common etiologies were subarachnoid hemorrhage (n = 20, 35.1%), ischemic stroke (n = 16, 28.1%), cerebral vein thrombosis (n = 6, 10.5%), and subdural hemorrhage (n=6, 10.5%). Bedside D-dimer demonstrated a sensitivity of 82.5% (95% CI, 70–91%) and specificity of 89.2% (95% CI, 87–91%) for identifying intracranial pathology, with a positive likelihood ratio of 7.6 (95% CI, 6.3–9.2) and negative likelihood ratio of 0.2 (95% CI, 0.1–0.35). Diagnostic accuracy was highest for **cerebral venous thrombosis**: sensitivity was 100% with a wide CI (95% CI, 54–100%), specificity was 86.8% (95% CI, 85–88%), and positive likelihood ratio was 7.6 (95% CI, 6.7–8.6). For **subarachnoid hemorrhage**, where sensitivity reached 90% (95% CI, 68–99%), specificity was 87.5% (95% CI, 86–89%), the positive likelihood ratio was 7.2 (95% CI: 5.9–8.8), and the negative likelihood ratio was 0.1 (95% CI: 0.03–0.4).

**Conclusion:**

Bedside D-dimer testing showed moderate performance as a screening adjunct in ruling out secondary headache due to intracranial causes in ED patients with non-traumatic headache and no neurological findings.

## INTRODUCTION

Headache is the fifth most common reason for emergency department (ED) presentations and a leading neurological cause worldwide, according to the Global Burden of Disease report.^1^,^2^ The International Headache Society classifies headache as primary and secondary.^3^ Patients diagnosed with primary headache generally require symptomatic management, whereas those with suspected secondary headache, particularly due to intracranial pathology, warrant further diagnostic evaluation in the ED, including cranial computed tomography (CT) and lumbar puncture when indicated. Although not all secondary headache disorders require urgent neuroimaging in the ED, pathologies such as subarachnoid hemorrhage (SAH) warrant urgent evaluation. Most ED patients with non-traumatic headache are ultimately diagnosed with primary headache; while the prevalence of serious secondary causes highly varies across regions, it is relatively low (overall 9.9%).

A secondary analysis of the multinational observational study, Headache in Emergency Departments, (N = 5,281) demonstrated substantial inter-regional variation in ED use of head CT for non-traumatic headache (28.9–46.6%), with even greater heterogeneity across hospitals within the same region. Diagnostic yield showed a similar pattern, ranging from 5.4% in Europe to 11.2% in Australia/New Zealand (and 9.1% in Colombia; 10.6% in Türkiye).^4^ Given the low prevalence but high clinical stakes of missing a secondary headache, clinicians face the challenge of safely and efficiently identifying which patients require urgent neuroimaging. A rapid, bedside biomarker with high sensitivity could assist in this decision-making process. Computed tomography entails exposure to ionizing radiation and, consequently, a non-trivial cancer risk. A recent *JAMA* study estimated that the 93 million CTs performed in the US in 2023 may lead to approximately 103,000 future cancers.^5^ Prudent imaging stewardship can reduce unnecessary radiation and downstream costs.

D-dimer is a protein degradation product generated by the breakdown of fibrinogen and fibrin during fibrinolysis. It is primarily used to rule out thromboembolic diseases, notably pulmonary embolism and deep vein thrombosis.^6^ Quantitative D-dimer assays exhibit high sensitivity for ruling out pulmonary embolism in appropriately selected ED patients; however, specificity is low because levels are frequently elevated in diverse conditions (eg, malignancy, bleeding disorders, pregnancy, and trauma), leading to false positives. The majority of secondary headache disorders are attributable to intracranial bleeding, tumors, or thrombotic processes, all of which are associated with elevated D-dimer levels. Therefore, D-dimer could function as an adjunctive screening tool, guiding the appropriate selection of patients for head CT. However, evidence evaluating this approach in patients presenting with non-traumatic headache is limited.

Our objective in this study was to evaluate the diagnostic performance of bedside D-dimer testing in identifying intracranial secondary causes of headache in ED patients presenting with non-traumatic headache and no neurological deficits.

## METHODS

### Study Setting

This was a secondary analysis of a prospective, multicenter cross-sectional study. which was conducted within an 18-month period in the EDs of six tertiary care hospitals in Türkiye. Each of the four EDs had an annual patient volume of 50,000, whereas the remaining two had 180,000. We obtained local ethical committee approval prior to the commencement of the study, and inform consent was provided by the study patients prior to recruitment.

**Population Health Research Capsule**

What do we already know about this issue?

*Detecting intracranial causes of non-traumatic headache in patients who have no neurological deficits is difficult and frequently results in unnecessary CT*.

What was the research question?

*Can bedside D-dimer detect intracranial causes in non-traumatic headache without neurologic deficit in the emergency department?*

What was the major finding of the study?

*D-dimer has a sensitivity of 82.5% (95% CI, 70–91%) and specificity of 89.2% (95% CI, 87–91%) for identifying any intracranial pathology*.

How does this improve population health?

*D-dimer has a moderate diagnostic performance for intracranial pathologies in non-traumatic headache without neurologic deficit that may result in unnecessary CT use*.

### Selection of Participants

Patients presenting with non-traumatic headache who were deemed eligible for cranial CT due to suspected intracranial pathology were prospectively included in this study. For this study, secondary headache was operationally defined as a headache directly attributable to intracranial pathology. This definition prioritizes the more urgent and critical clinical implications of intracranial causes over extracranial etiologies such as sinusitis or glaucoma. Furthermore, since the study patients had to be without neurological deficits, all demonstrated normal mental status and normal findings on neurological examination. The neurological examination included assessment of mental status, lateralizing motor or sensory deficits, speech abnormalities, cranial nerve function, and cerebellar function. The decision to perform a CT was made by the attending physician based on their clinical judgment and adherence to the study’s inclusion and exclusion criteria, thereby reflecting pragmatic, real-world ED practice. Patient recruitment was conducted continuously, 24 hours a day, seven days a week.

### Exclusion Criteria

We excluded patients who met any of the following criteria:

- Recent head trauma (within the prior week)
- < 18 years of age
- Presence of neurological deficits
- Pregnancy
- Fever
- A known diagnosis of primary brain tumor or metastatic brain lesions
- Hematologic conditions, including aplastic anemia, lymphoma, or idiopathic thrombocytopenic purpura
- History of recent neurosurgery or hydrocephalus
- Refusal to provide informed consent
- History of deep vein thrombosis or pulmonary embolism.

### Data Collection

Emergency medicine residents collected the data by completing a standardized study form. This form included demographic characteristics of the patients, inclusion and exclusion criteria, and several headache-related features, such as sudden onset, history of similar headaches, “worst-ever” headache, associated vomiting, and syncope. The patient’s response to analgesics was also recorded. However, the analgesics administered were not standardized; both the choice of agent and the dosing regimen were determined at the discretion of the attending physician. The chart abstractors were not blinded to the study hypothesis.

Emergency physicians ordered CT without contrast, which has been shown to be cost saving in patients presented to the ED with acute non-traumatic symptoms referable to the brain. The decision to perform contrast-enhanced CT was made by radiologists based on clinical findings, non-contrast CT results, and differential diagnoses such as tumors or venous thrombosis. This was also true for performing a CT angiography. The CTs were reviewed by the radiology department either by an attending physician or a senior resident. Treating physicians were not restricted to perform additional tests such as lumber puncture for the final diagnosis.

### D-dimer Measurement

Whole blood D-dimer levels were quantitatively assessed using the Triage D-dimer test device (Biosite Diagnostics Inc., San Diego, CA). This diagnostic tool employs microcapillary fluidics and a fluorescence immunoassay (using the 3B6 antibody) to determine D-dimer concentrations in whole blood or plasma samples. The analysis is fully automated and performed on a portable fluorometer. Results were reported in nanograms per milliliter (ng/mL) (D-dimer calibrated), with a predefined cutoff value of 500 ng/mL. Measurements and interpretation of the D-dimer levels were performed by the physicians who recruited the study patients and were also responsible for treating them. Thus, the treating physicians were not blinded to the D-dimer levels. It should be noted that measurements of D-dimer levels were done before a decision was made to perform CT. The radiologists who interpreted the images were blinded to the D-dimer levels.

### Primary Outcome

The **primary outcome** of this study was defined as the presence of any **intracranial cause of headache**, including intracranial hemorrhages such as SAH, subdural hemorrhage and intraparenchymal hemorrhage, cerebral venous thrombosis, brain tumors, ischemic stroke, meningitis, and encephalitis. Extracranial causes of headache, such as sinusitis or mastoiditis, even if detected on head CT, were not considered primary outcomes. In addition to the final diagnosis made through CT findings (instant findings) and additional tests in the ED, a **one-month telephone follow-up** was conducted with patients after their ED visit to identify any alternative diagnoses made during that period. At each participating center, a study physician conducted the follow-up calls.

### Statistical Analysis

We analyzed study data using SPSS 23.0 (IBM Corporation, Armonk, NY) 23.0 and MedCalc for Windows, v23.3.7 (MedCalc Software, Ostend, Belgium). The numeric data were expressed by mean and standard deviation and frequent data as rates. We reported the diagnostic value of bedside D-dimer levels by sensitivity, specificity, and likelihood ratios along with 95% confidence intervals, which we used to present the confidence estimates of each finding. Receiver operating characteristic (ROC) curve analysis was performed to calculate the area under the curve (AUC). All hypotheses were constructed as two tailed, and a critical value of 0.05 was accepted as significant.

## RESULTS

**Figure 1:**
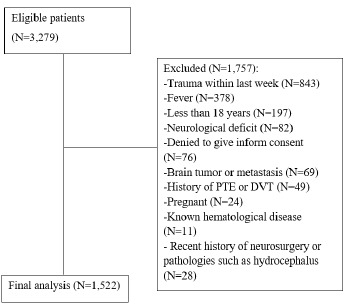
Patient flowchart in study evaluating the diagnostic utility of bedside D-dimer testing for intercranial causes of non-traumatic headache.

Of 3,279 eligible patients, 1,757 were excluded, resulting in 1,522 patients included in the final analysis (see Figure 1). A total of 104 (6.8%) patients could not be reached at one-month telephone follow-up. The mean age of the study participants was 47.6±16.8years, and 643 (42.2%) were male.

Regarding headache characteristics, 762 patients (50.1%) reported an abrupt onset of pain, 545 (35.9%) experienced similar previous headaches, 92 (6.1%) presented with syncope, and 71(4.7%) noted aggravation with physical activity. Additionally, 276 patients (18.1%) reported an analgesic response to their headache (Table 1).

### Secondary Headache Diagnoses

A total of 57 patients (3.7%) were diagnosed with an intracranial pathology. The most prevalent causes of secondary headache were SAH in 20 patients (35.1%), ischemic stroke in 16 patients (28.1%), six patients with subdural hemorrhage (10.5%), six with cerebral venous thrombosis (10.5%), and five with brain mass (8.8%) (Table 2).

While D-dimer had a sensitivity of 82.5% (95% CI, 70–91%) and specificity of 89.2% (95% CI, 87–91%) for predicting any kind of secondary headache, it performed better in patients with intracranial bleeding (sensitivity: 89.3%, 95% CI, 72–98%; specificity: 87.9%, 95% CI, 86–90%) and SAH (sensitivity: 90%, 95% CI: 68–99%; specificity: 87.5%, 95% CI, 86–89%,) and cerebral venous thrombosis (sensitivity: 100%, 95% CI, 54–100%; specificity: 86.8%, 95% CI, 85–88%) (Table 3). The patient with encephalitis had a D-dimer value of 860 ng/mL, while the patient with meningitis had a value of 100 ng/mL.

ROC analysis revealed an AUC value of 0.901 (95% CI: 0.885 to 0.915), indicating good diagnostic validity.

## DISCUSSION

This study suggests that D-dimer possesses moderate diagnostic utility in predicting intracranial pathology among headache patients classified as low risk for a secondary cause. Diagnostic performance was highest for cerebral venous thrombosis and, to a lesser extent, for intracranial hemorrhage, including SAH.

There remains a scarcity of high-quality studies supporting the use of biomarkers for risk stratification in patients presenting with headache.^7^

A systematic review and meta-analysis by Dentali et al^8^ investigated the diagnostic utility of D-dimer in cerebral venous thrombosis, reporting a bivariate weighted mean sensitivity and specificity of 93.9% and 89.7%, respectively. However, only six of the included studies were prospective and involved patients specifically suspected of having cerebral venous thrombosis, and the studies were generally characterized by small sample sizes. A recent meta-analysis by Alons et al^9^ pooled three studies of patients with isolated headache, which is similar to our study, and combined them with the authors’ own dataset. The pooled diagnostic accuracy of D-dimer for identifying cerebral venous thrombosis was 97.8% sensitivity and 84.9% specificity. In the present study, while sensitivity for detecting cerebral venous thrombosis was 100% the 95% CI was wide, reflecting the small number of events and limiting the precision of this estimate. By contrast, specificity was moderate but more precisely estimated at 86.8% (95% CI, 85–88%). These findings should be considered hypothesis-generating and warrant confirmation in larger, independent cohorts.

D-dimer has a moderate diagnostic utility in patients with ischemic stroke with a sensitivity and specificity of 81.3% and 87.2%, respectively, according to our study results. A systematic review by Haapaniemi et al reported on studies with regard to D-dimer levels and ischemic stroke.^10^ The studies had small sample sizes and were, thus, prone to random error. Although D-dimer levels were higher than in the healthy controls in these studies, D-dimer was within normal limits (< 500 ng/ml) in most studies, thereby preventing an exact conclusion concerning the issue. A more recent meta-analysis reported higher D-dimer levels in stroke patients but with significant heterogeneity.^11^

According to the results of our study, D-dimer exhibits a sensitivity of 89.3% and a specificity of 87.9% in diagnosing intracranial hemorrhage. These values are largely consistent for SAH, with a sensitivity of 90% and a specificity of 87.5%. These findings align with previous research by Fujii et al^12^ and Delgado et al,^13^ who also reported elevated D-dimer levels in patients with intracranial hemorrhage compared to healthy controls. Peltonen et al^14^ observed elevated D-dimer levels in a small cohort of 25 SAH patients compared to seven healthy controls. Similarly, a meta-analysis by Zhou et al,^15^ comprising 13 studies, reported higher D-dimer levels in patients with intracerebral hemorrhages than in control groups. However, this meta-analysis was limited by small sample sizes and significant heterogeneity among the included studies. As a product of the coagulation system, D-dimer levels are expected to rise in patients experiencing bleeding. Despite D-dimer demonstrating relatively good sensitivity and specificity in the context of intracranial hemorrhage, false-negative and false-positive results can occur, potentially influenced by the volume and size of the bleeding. In line with this, Fujii et al reported a direct correlation between increases in D-dimer levels and the severity of both intracranial hematoma and SAH.

Overall, the medical literature provides limited high-quality evidence supporting the use of D-dimer as a screening adjunct in patients presenting to the ED with non-traumatic headache. Non-traumatic headache patients without neurological deficits are of particular concern, as they pose a notable diagnostic challenge for emergency clinicians. D-dimer may offer adjunctive value in this population; however, further studies are needed to determine whether its use reduces unnecessary cranial CT and improves patient-centered outcomes.

Receiver operating characteristic analysis revealed an area under the curve value of 0.901 (95% CI, 0.885–0.915), which indicates a good diagnostic validity.

## LIMITATIONS

We used a point-of-care D-dimer assay with a prespecified cutoff of 500 ng/mL, consistent with a widely adopted clinical threshold. Although the manufacturer recommends 400 ng/mL, prior evaluation of the Triage assay reported no clinically meaningful difference in diagnostic performance when using a 500 ng/mL cut-off. However, the overall concordance of the D-dimer test was 89.3% when compared to the AxSYM D-dimer (Abbott Diagnostics, Abbott Park, IL), with a kappa value of 0.68.^16^ This finding suggests the need for replication of the present study’s findings using alternative D-dimer measurement methodologies. In addition to the measurement technique of D-dimer, these measurements were conducted by the recruiting physician who were not blinded to the D-dimer levels. Hence, it may have led to observer bias. Moreover, the chart abstractors were not blinded to the study hypothesis or to the D-dimer levels.

In this study we could not infer the validity of D-dimer for diagnosing potential intracranial infectious causes of secondary headache, primarily due to the exclusion of patients presenting with fever and altered mental status. Notably, two patients diagnosed with meningitis and encephalitis were identified through one-month telephone follow-up. The utility of D-dimer in the context of infectious etiologies of headache warrants further investigation in future research.

Another limitation to this study was the absence of a standardized workflow for the enrolled patients. Although this approach was more pragmatic and reflected real-world clinical practice, it may have led to selection bias and may have resulted in missed intracranial pathologies compared to a structured workflow. Nevertheless, this limitation was likely mitigated by the one-month telephone follow-up. Additionally, to precisely determine the diagnostic value of D-dimer in our study population, we opted to exclude patients with conditions known to increase D-dimer levels, with the exception of extracranial solid malignancies without intracranial metastasis. Although 12 patients had extracranial solid malignancies, a re-analysis conducted after their exclusion yielded only minimal changes in the results, which were insignificant. The stringent exclusion of numerous pathologies related to elevated D-dimer levels may restrict the applicability of these findings in real-world ED settings. Consequently, future research should focus on more pragmatic trials including pathologies related to increased D-dimer levels to enhance the generalizability of these results.[Fig f2-wjem-27-304]

A total of 104 (6.8%) patients could not be reached for telephone follow-up at the one-month mark. Consequently, we could not definitively ascertain whether any secondary headache diagnoses were missed within this specific cohort, which represents an additional potential limitation of the study.

Furthermore, we did not conduct an *a priori* sample size calculation before the study, which may have resulted in insufficient power, limiting the precision of estimates and the strength of inferences. Nonetheless, the relatively large sample size strengthens the stability of the estimates and lends credibility to the results.

Another limitation is the heterogeneity of analgesic regiments, which may have influenced symptom-based clinical assessment and, in turn, head CT use. Another limitation that may have influenced the decision to perform a CT is that D-dimer levels were measured prior to the imaging decision. Although the emergency physicians enrolling the patients were not accustomed to using D-dimer as an ancillary marker for CT indication in headache patients, its availability could still have influenced their clinical judgment.

## CONCLUSION

In patients presenting with non-traumatic headache and no concomitant neurological deficits, D-dimer provides a moderate level of diagnostic utility for detecting any intracranial pathology. However, its diagnostic performance is particularly superior in ruling out cerebral venous thrombosis, followed by subarachnoid hemorrhage. The diagnostic validity of D-dimer in specific subgroups with non-traumatic headache should be a focus of future investigation.

## Figures and Tables

**Figure 2 f2-wjem-27-304:**
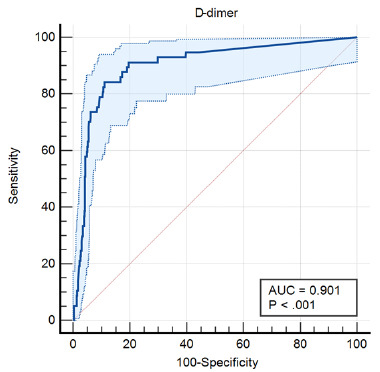
Receiver operating characteristic curve displaying the diagnostic value of D-dimer in any intracranial pathology. *AUC*, area under the curve

**Table 1:** Demographic features of study patients who presented to the emergency department with non-traumatic headache and underwent head computed tomography.

Variable	N = 1,522
Age, mean±SD	47.6±16.8
Sex (Male)	643 (42.2)
Abrupt onset of pain	762 (50.1)
Alleviated with analgesic	276 (18.1)
History of similar headaches	545 (35.8)
Syncope	92 (6)
Aggravated by physical activity	71 (4.7)
Vomiting	426 (28)
Worst headache ever	901 (59.2)

* All data presented as frequencies and rates, unless stated otherwise.

*CT*, computed tomography; *ED*, emergency department.

**Table 2:** Prevalence of pathologies related to secondary headache in study patients.

Variable	N = 57
Subarachnoid hemorrhage	20 (35.1)
Subdural hemorrhage	6 (10.5)
Brain mass	5 (8.8)
Ischemic stroke	16 (28.1)
Cerebral venous thrombosis	6 (10.5)
Intraparenchymal hemorrhage	2 (3.5)
Meningitis	1 (1.8)
Encephalitis	1 (1.8)

* All data presented as frequencies and rates, unless stated otherwise.

**Table 3:** Diagnostic value of bedside D-dimer in various kinds of secondary headaches in the emergency department.

Secondary headaches	Sensitivity (95% CI)	Specificity (95% CI)	Positive likelihood ratio (95% CI)	Negative likelihood ratio (95% CI)
Any intracranial pathology	82.5 (70 to 91)	89.2 (87 to 91)	7.6 (6.3 to 9.2)	0.2 (0.1 to 0.35)
Intracranial bleeding*	89.3 (72 to 98)	87.9 (86 to 90)	7.4 (6 to 8.9)	0.12 (0.04 to 0.4)
SAH	90 (68 to 99)	87.5 (86 to 89)	7.2 (5.9 to 8.8)	0.1 (0.03 to 0.4)
Ischemic stroke	81.3 (54 to 96)	87.2 (85 to 89)	6.3 (4.8 to 8.3)	0.2 (0.08 to 0.6)
Cerebral venous thrombosis	100 (54 to 100)	86.8 (85 to 88)	7.6 (6.7 to 8.6)	0
Brain mass	40 (5 to 85)	86.5 (85 to 88)	3 (1 to 9)	0.7 (0.3 to 1.4)

* Including SAH, subdural hemorrhage, and intraparenchymal hemorrhage

*SAH*, subarachnoid hemorrhage.
